# Clinical trial registries: more international, converging efforts are needed

**DOI:** 10.1186/s13063-017-1836-4

**Published:** 2017-02-27

**Authors:** Claudia Pansieri, Chiara Pandolfini, Maurizio Bonati

**Affiliations:** Department of Public Health, Laboratory for Mother and Child Health, IRCCS-Istituto di Ricerche Farmacologiche “Mario Negri”, Via Giuseppe la Masa 19, 20156 Milan, Italy

**Keywords:** Clinical trials as Topic, Databases, Factual, Registries, Transparency, Website usability

## Abstract

Clinical trial registries are being increasingly acknowledged worldwide. We searched for possibly trustworthy online registries that are not already included in the International Clinical Trials Registry Platform to evaluate whether other useful trial data sources exist and whether they could potentially be consulted, since the strategy search within this platform has recently been questioned. Fifty-nine registries were initially identified, and 11 of them fit the criteria applied and were analyzed for quality and usability. Four additional, potentially reliable registries were identified that researchers could exploit in order to obtain a more global view of the issue being investigated.

## Background

Clinical trial registries are being increasingly acknowledged worldwide. Many registries focusing on specific populations, conditions, or interventions exist, and were developed to meet different needs. Currently, policymakers and the scientific community are moving, albeit not without concern, towards the registration of almost all trial-related information [1].

Despite the fact that multiple mandates have brought many countries to establish their own regional, national, or international registries, many trials remain unregistered or registered retrospectively or with poor quality information [2].

Given that the registration of trials is deemed the first step in the transparency process, ten years ago the World Health Organization launched the International Clinical Trials Registry Platform (ICTRP), which attempted to harmonize the information originating from these different registries, generating a more efficient strategy to search for trials. Although this led to a more realistic picture of the number of trials being carried out worldwide, trial coverage of the ICTRP has recently been questioned [3, 4]. In this context, characterized by uncertainty in finding all currently registered clinical trials, we searched for additional online clinical trial registries in order to assess whether other useful sources of trial data, beyond the 15 ICTRP platform’s registries, exist and whether they could potentially be consulted. In particular, our aim was to provide researchers wishing to find trials on a specific disease or drug with additional, solid data sources.

## Main text

We searched Medline and Embase for papers published up to July 2015, and Google for direct links to registries (details are presented in Table 1). We excluded from analysis registries that were already part of the ICTRP, were not freely accessible, or were limited to specific therapeutic areas. We further limited resulting registries to those (1) whose data was original, i.e., not taken from another registry, (2) that did not simply provide a title and refer users to a different registry for additional data, (3) with English language webpages and trial data, (4) that were not aimed solely at patient recruitment, (5) that provided at least minimal search options or separate data fields describing the intervention or drug, thus permitting at least a visual search through these two fields, and (6) that provided sufficient trial details.

**Table 1:** Medline, Embase, and Google search strategies

The Medline search strategy involved combinations of free text terms with search terms chosen from the database’s Medical Subject Headings (MeSH) and included: “internet”[Mesh] AND ((“registries”[Mesh] OR registries[Title/Abstract]) AND (trial[Title/Abstract] OR trials[Title/Abstract]). Similarly, the Embase search was based on the terms chosen from the Emtree thesaurus: (‘register’/exp AND ‘internet’/exp AND ‘clinical study’/exp). The Google search, aimed at finding links concerning registries, involved the same keywords as the database searches, in addition to several synonyms. The bibliographies of selected articles were examined to identify additional relevant studies, and several websites were searched for links to additional registries.	

Two of the authors performed a quality analysis of the selected registries by checking for the presence of pre-selected items concerning mainly quality and usability (see Table 2), and discrepancies were discussed and resolved with the third author.

**Table 2:** Descriptive characteristics and quality and usability criteria applied to the 11 selected registries

Name of registry	Health Canada	Weill Cornell	Yale	Mayo Clinic	HKClinical Trials	NMRR	HAS	SUKL	REec	NIHR-CRN	Mario Negri
Descriptive											
Country	Canada	USA	USA	USA	Hong Kong	Malaysia	Singapore	Czech Republic	Spain	UK	Italy
Year of setup	2013	2013	N/A	N/A	2005	2007	2012	2007	N/A	N/A	N/A
Governance: funding agency	Government	N/A	N/A	N/A	University	Government	Government	Government	Government	Government	Non profit organization
Governance: managing agency	Government	Medical college/ Hospital	University	Non profit organization	University	Government	Government	Government	Government	Government	Non profit organization
The registration has some limitation?	Yes	Yes	Yes	Yes	No	Yes	Yes	Yes	Yes	Yes	Yes
*Type of study (RCT-observational-pharmacological…)*	Yes	N/A	N/A	No	No	No	No	Yes	No	N/A	No
*Geographic (sponsor or where conducted)*	Local	Local	Local	Local	No	Local	Local	Local	Local	Local	Local
Shows total number of trials registered	No	No	Yes (281)^b^	No	Yes (1845)^b^	Yes (7311)^b^	Yes (558)^b^	No	Yes (1253)^b^	Yes (15,807)^a^	Yes (165)^b^
Shows total number of ongoing trials registered	No	No	No	No	Yes	No	Yes	Yes	Yes	No	No
Shows total number of pediatric trials registered	No	No	No	No	No	No	No	Yes	Yes	No	No
Quality											
Does the registry have a section for the results?	No	No	No	No	No	No	No	No	No	No	No
Does the registry provide trial protocol?	No	No	No	No	No	No	No	No	No	No	No
Does the registry have a distinct pediatric trials section (a flag or a therapeutic area pediatric…)?	Yes	No	No	No	No	No	No	Yes	Yes	No	No
Report the date of the last webpage update	Yes	No	No	No	No	No	Yes	Yes	No	No	No
Report the date of the last trial update	No	No	No	No	No	No	No	No	No	No	No
Clinical trial data output (RSS, XML or CVS)	No	No	No	No	No	No	No	No	No	Yes	No
Possibility to contact the registry team	Yes	Yes	Yes	No	Yes	No	Yes	No	Yes	Yes	Yes
Search tips	Yes	No	No	Yes	Yes	Yes	No	No	Yes	No	No
Usability											
Multilingualism of the website	Yes	No	No	No	No	No	No	Yes	Yes	No	Yes
Multilingualism of clinical trial information	Yes	No	No	No	No	No	No	Yes	Yes	No	Yes
Simple search option	Yes	Yes	Yes	Yes	Yes	Yes	Yes	Yes	Yes	Yes	Yes
*Free text*	Yes	Yes	Yes	Yes	Yes	Yes	Yes	Yes	Yes	Yes	Yes
*List of therapeutic area or disease*	No	Yes	Yes	No	No	No	Yes	Yes	No	Yes	No
Elaborate search options	Yes	Yes	Yes	Yes	Yes	Yes	Yes	Yes	Yes	Yes	Yes
*Keywords*	No	No	No	No	Yes	No	No	No	No	No	Yes
*Status*	Yes	No	No	Yes	Yes	Yes	Yes	Yes	Yes	No	No
*Study type*	No	Yes	No	No	Yes	Yes	No	No	No	No	No
*Intervention/drug*	Yes	Yes	No	No	Yes	No	Yes	No	Yes	No	Yes
*Condition/disease*	Yes	No	No	No	Yes	No	No	No	Yes	No	Yes
*Phase*	No	Yes	No	Yes	Yes	No	Yes	No	Yes	No	No
*Age*	Yes	No	Yes	Yes	No	No	No	Yes	Yes	No	No
*Gender*	Yes	No	Yes	Yes	No	No	No	Yes	Yes	No	No
*Location*	No	No	No	Yes	No	No	Yes	Yes	Yes	No	No
*Sponsor*	Yes	No	No	No	Yes	Yes	Yes	No	Yes	No	No

^a^Number may exceed total number of trials in the registry because some trials may be represented in more than one category. This numbers refers to the total number of trials registered before July 2015

^b^The total number of trials registered and rechecked was updated in September 2016: Yale - 459; HKClinical trials - 1999; NMRR - 8890; HAS - 574; REec - 2568;

Mario Negri - 178

URLs: Health Canada: http://ctdb-bdec.hc-sc.gc.ca/ctdb-bdec/index-eng.jsp; Weill Cornell: http://jcto.weill.cornell.edu/; Yale: http://www.yalestudies.org/; Mayo Clinic: http://www.mayo.edu/research/clinical-trials; HKClinicalTrials: http://www.hkclinicaltrials.com/; NMRR: https://www.nmrr.gov.my/fwbLoginPage.jsp; HAS: http://www.hsa.gov.sg/content/hsa/en/Health_Products_Regulation/Clinical_Trials/Overview/Clinical_Trials_Register.html; SUKL: http://www.sukl.eu/modules/evaluation/; REec: https://reec.aemps.es/reec/public/web.html; NIHR-CRN: http://public.ukcrn.org.uk/search/ "The NIHR-CRN is no longer active and users can visit the UK Clinical Trials Gateway (https://www.ukctg.nihr.ac.uk/) to search for trials."; Mario Negri: http://registro.marionegri.it/frontend/index.php

A total of 59 registries were initially identified: 28 in Europe, 22 in North America, 5 in Asia, and 2 in both Oceania and South America. When filtered based on the above-mentioned criteria, only 11 registries resulted (Table 2), and the analysis of the pre-selected items had a good inter-rater agreement (90% agreement, kappa = 0.79).

Concerning quality and usability, the option to limit a search by trial status (e.g., ongoing versus recruiting) was only provided by 7/11 registries, while multilingualism of the trial data was a characteristic in four of the seven non-solely-English speaking countries. None of the registries track study changes or the date of last update of each trial record, show additional study identifiers, or provide links to publications. In general, the registries fulfilled few of the quality criteria.

Overall, 6/11 registries were public service registries funded and managed by public entities. Limitations on the trials accepted for registration were mostly related to the country (or research institute) of origin of the trials, because most of the registries were local. None explicitly stated that observational studies could be registered. The search options provided varied, and only 6 registries permitted the user to see, or calculate, the total number of registered trials (Table 2). This number was checked again in September 2016, before publication, and updated values are listed in a footnote to Table 2. Regrettably, none of the registries had a section for trial results.

Four of the clinical trial registries (HKClinicalTrials, NMRR, REec, Mario Negri) provide data that fulfill the list of pre-selected items on quality and usability (e.g., randomization, blinding, inclusion/exclusion criteria), resulting as more aligned, in terms of transparency, with the ICTRP’s 15 registries.

## Conclusions

Transparent reporting of clinical trials is vital for decision making. Registries, initially designed to store only basic information, will soon likely be modified and expanded to hold additional data, such as clinical study reports and individual patient data, and will evolve to become a point of reference for evidence-based medicine [5, 6].

The greatest achievement of the ICTRP is that any investigator, from any region of the world, can easily and freely consult the “portfolio” of trials registered in any of the approved registries. The real number of trials conducted all over the world, however, remains unknown, and the fact that searching the ICTRP as a whole yielded fewer results than searching the individual trial registries led us to this search [3, 4].

Identifying registries that collect original data and that are not already included in the ICTRP platform was difficult to perform, and the most efficient search strategy was the Google search. We found that, except for countries such as Iran, South Africa, Brazil, and Cuba, registries have been set up almost exclusively by higher income countries. Some areas of the globe, such as the Middle East, Africa, and Latin America, representing many lower income countries, still do not have locally or nationally representative registries. Researchers have found that the establishment, usually on a voluntary basis, of national and local-language registries has a significant impact on the proportion of trials registered in a country [7]. Considering that a large proportion of clinical trials may involve low- to lower-middle income countries [8, 9], and that this percentage is expected to grow following the increasing worldwide initiatives , this may represent an essential data “incubator” for the global knowledge base. Mandates are still lacking in most areas of the world [7], and the lack of systematic approaches results in a dispersal of information and resources. Several countries, however, do not have enough resources or have limited Internet access [10], and their only way to make their research public is through existing international registries.

Registries identified through our study differ in size and search options provided, permitting different searching sensitivities and leading to differences in completeness of results. Our assessment shows that four additional, potentially solid sources of trial data may exist that researchers could exploit in order to obtain a more global view of the issue being investigated. Two of the registries analyzed, however, have been modified in the last few months. The Spanish registry, REec, has been improved, and the NIHR’s Portfolio database (NIHR-CRN) is now provided through the UK Clinical Trials Gateway website (https://www.ukctg.nihr.ac.uk/) and no longer fits our selection criteria. Possible changes to the sources of trial data should be considered specifically in a future, ad hoc evaluation of the reliability and accuracy of these sources, as this topic was beyond the scope of this commentary.

The information held in the registries has many audiences, from patients to health care providers, researchers, pharmaceutical companies, and regulators. Their role in systematic reviews is also being increasingly recognized [11]. The setting up of local/national registries is fundamental for making data more accessible locally, e.g., in a country’s own language, but cannot be fully exploited, for example by the ICTRP, due to lack of homogeneity in quantity and quality of data provided. The proliferation of registries inevitably imposes a bigger regulatory burden due to the need for standardization of information, greater usability and transparency, and routine checks for completeness and internal consistency.

The ICTRP represents a potent effort towards data accessibility, albeit with room for improvement, both in searching capacity and in trial coverage [3, 4]. The four registries we identified, whose greater usability and data quality are more aligned with those of the ICTRP’s registries, could potentially be considered for inclusion in its platform. Unless worldwide legislation is put into place, however, establishing homogeneous criteria in trial documentation, and allowing registries to become common information sources, even this enormous effort will remain limited in its potential.

## References

[1] Taichman DB, Backus J, Baethge C (2016). Sharing clinical trial data: a proposal from the International Committee of Medical Journal Editors. PLoS Med.

[2] Pansieri C, Pandolfini C, Bonati M (2015). The evolution in registration of clinical trials: a chronicle of the historical calls and current initiatives promoting transparency. Eur J Clin Pharmacol.

[3] Glanville JM, Duffy S, McCool R (2014). Searching ClinicalTrials.gov and the International Clinical Trials Registry platform to inform systematic reviews: what are the optimal search approaches?. J Med Libr Assoc.

[4] Munch T, Dufka FL, Greene K (2014). RReACT goes global: perils and pitfalls of constructing a global open-access database of registered analgesic clinical trials and trial results. Pain.

[5] Eichler HG, Abadie E, Breckenridge A (2012). Open clinical trial data for all? A view from regulators. PLoS Med.

[6] Zarin DA, Tse T (2016). Sharing individual participant data (IPD) within the context of the trial reporting system (TRS). PLoS Med.

[7] Viergever RF, Li K. Trends in global clinical trial registration: an analysis of numbers of registered clinical trials in different parts of the world from 2004 to 2013. BMJ Open. 2015;5(9):e008932 10.1136/bmjopen-2015-008932 PMC4593134 26408831

[8] Ghersi D, Pang T (2008). En route to international clinical trial transparency. Lancet.

[9] Richter TA. Clinical research: a globalized network. PLoS One. 2014;9(12):e115063. 10.1371/journal.pone.0115063 PMC4269395 25517976

[10] Siegfried N, Volmink J, Dhansay A. Does South Africa need a national clinical trials support unit? S Afr Med J. 2010;100(8):521–4. 10.7196/samj.3958 20822621

[11] Baudard M, Yavchitz A, Ravaud P, et al. Impact of searching clinical trial registries in systematic reviews of pharmaceutical treatments: methodological systematic review and reanalysis of meta-analyses. BMJ. 2017;356:j448. 10.1136/bmj.j448 PMC5421496 28213479

